# Reactive wear protection through strong and deformable oxide nanocomposite surfaces

**DOI:** 10.1038/s41467-021-25778-y

**Published:** 2021-09-17

**Authors:** Chang Liu, Zhiming Li, Wenjun Lu, Yan Bao, Wenzhen Xia, Xiaoxiang Wu, Huan Zhao, Baptiste Gault, Chenglong Liu, Michael Herbig, Alfons Fischer, Gerhard Dehm, Ge Wu, Dierk Raabe

**Affiliations:** 1 Max-Planck-Institut für Eisenforschung, Max-Planck-Straße 1, 40237 Düsseldorf, Germany; 2 School of Materials Science and Engineering, Central South University, Changsha, 410083 China; 3 State Key Laboratory of Powder Metallurgy, Central South University, Changsha, 410083 China; 4 Department of Mechanical and Energy Engineering, Southern University of Science and Technology, Shenzhen, China; 5 Department of Mechanical Engineering, City University of Hong Kong, Hong Kong, China; 6 School of Metallurgical Engineering, Anhui University of Technology, Maanshan, 243000 China; 7 Shagang School of Iron and Steel, Soochow University, Suzhou, 215137 China; 8 Department of Materials, Royal School of Mine, Imperial College London, London, SW7 2AZ UK

**Keywords:** Engineering, Mechanical properties, Metals and alloys

## Abstract

Wear-related energy and material loss cost over 2500 Billion Euro per year. Traditional wisdom suggests that high-strength materials reveal low wear rates, yet, their plastic deformation mechanisms also influence their wear performance. High strength and homogeneous deformation behavior, which allow accommodating plastic strain without cracking or localized brittle fracture, are crucial for developing wear-resistant metals. Here, we present an approach to achieve superior wear resistance via in-situ formation of a strong and deformable oxide nanocomposite surface during wear, by reaction of the metal surface with its oxidative environment, a principle that we refer to as ‘reactive wear protection’. We design a TiNbZr-Ag alloy that forms an amorphous-crystalline oxidic nanocomposite surface layer upon dry sliding. The strong (2.4 GPa yield strength) and deformable (homogeneous deformation to 20% strain) nanocomposite surface reduces the wear rate of the TiNbZr-Ag alloy by an order of magnitude. The reactive wear protection strategy offers a pathway for designing ultra-wear resistant alloys, where otherwise brittle oxides are turned to be strong and deformable for improving wear resistance.

## Abstract (web summary)

Wear-resistant metals have long been a pursuit of reducing wear-related energy and material loss. Here the authors present the ‘reactive wear protection’ strategy via friction-induced in situ formation of strong and deformable oxide nanocomposites on a surface.

## Introduction

Wear-related energy loss and component damage, including friction and remanufacturing of components that failed by wear contacts, accounts for 23% of the global energy consumption with an estimated cost of over 2500 Billion Euro per year^1^. Since metals are the most used engineering materials, wear-resistant metals have long been the pursuit in materials design, yet are challenging to achieve^2^. According to classical wear theory, a material with higher strength exhibits higher wear resistance due to a reduced contact area at a fixed stress^3^. High-strength martensitic steels^4^ and cobalt-based alloys^5^ have been extensively used in environments with severe wear. Nanograined metals with higher strength than their coarse-grained counterparts have been developed for achieving improved wear resistance^6^. However, there exist conflicting reports about the wear performance of nanograined metals^7^. It has been found that grain growth or grain boundary sliding/grain rotation mechanisms (following the inverse Hall-Petch relation^8^) can reduce wear resistance^9^. The wear resistance of nanograined metals can be enhanced if their structural evolution upon wear promotes homogeneous plastic deformation, which prevents strain softening and brittle fracture^10^. Therefore, the design of wear-resistant metals requires a high strength in conjunction with appreciable homogeneous deformation ability upon wear. Gradient nanograined metals offer enhancement in wear resistance compared to their nanograined counterparts, due to the suppression of inhomogeneous plastic deformation at the contact surfaces, restricting sliding-induced cracking and localized brittle fracture^11^. Amorphous alloys, or metallic glasses (MGs), lack crystalline structures and usually possess improved yield strengths (*σ*_*y*_~*E*/50, *E* is elastic modulus) compared to their crystalline counterparts^12^. Nevertheless, the plastic deformation of MGs is highly localized in shear bands, which results in cracks and brittle wear particles, thus evoking a micro-cutting mechanism which dramatically accelerates wear^13^. The plastic deformability of MGs can be enhanced when introducing chemical or topological heterogeneity to generate multiple shear bands, e.g., by introducing glass-glass interfaces (nanoglasses)^14^, a secondary amorphous phase (dual-phase MGs)^15^, or a crystalline phase (amorphous-crystalline composites)^16,17^. However, the shear banding response of the amorphous phase still limits wear resistance^18^. Surface oxides, with a nanocomposite structure comprising crystalline oxide nanoparticles embedded in an amorphous matrix, can be formed on steels^19^ and cobalt-based alloys^20^ during wear. It has been reported that nanocomposites containing brittle crystalline phases usually reveal inhomogeneous plastic deformation due to shear banding^21^. If the brittle crystalline phase (such as crystalline oxide nanoparticles) in the nanocomposite is replaced by a ductile solid solution crystalline phase, homogeneous plastic deformation can be achieved^22^.

Here, we propose an alloy design concept based on an enthalpy-guided approach to promote formation of solid solution nanocrystals, rather than crystalline oxide nanoparticles, in the amorphous matrix during wear. This structure enables a high strength and homogeneous plastic deformation of the friction-induced oxidic nanocomposite surface layer. We realize this concept in a multi-component (TiNbZr)_75_Ag_25_ (at.%) alloy, by blending a TiNbZr medium-entropy alloy with Ag to create the desired self-lubricating nanostructure upon wear exposure. A ball-on-disk sliding test at a maximum contact stress of 1.0 GPa reveals that the (TiNbZr)_75_Ag_25_ alloy exhibits a friction coefficient of around 0.09 in air. The tribological contact introduces environmental O into the alloy, facilitating new atomic coordination with large negative enthalpy of mixing, which enhances the alloy’s glass-forming ability^23^. Besides, Ag has a positive enthalpy of mixing with Nb (+16 kJ/mol)^24^, which serves as a thermodynamic driving force for the nucleation of Ag nanocrystals from the amorphous matrix. Consequently, a ~400 nm thick amorphous-crystalline nanocomposite layer, with ~10 nm-sized Ag nanocrystals embedded in an amorphous oxide matrix, forms on the crystalline alloy surface upon wear in air. This surface nanocomposite exhibits a yield strength of 2.4 GPa and homogeneous deformation to 20% strain, as revealed by nano-pillar compression tests at ambient temperature. The formation of a strong and deformable oxide nanocomposite surface promotes an ultralow wear rate of the (TiNbZr)_75_Ag_25_ alloy, which is two orders of magnitude lower than that of a TiNbZr alloy exposed to identical test conditions, and is an order of magnitude lower than that of a (TiNbZr)_75_Ag_25_ alloy tested in Ar atmosphere. This represents a strategy for achieving exceptional wear resistance by utilizing the structural and chemical evolution of metal surfaces through alloying them not during synthesis but in-situ during wear-exposure with environmental O. We refer to this principle as ‘reactive wear protection’.

## Results

### Structure and composition characterizations

**Fig. 1:**
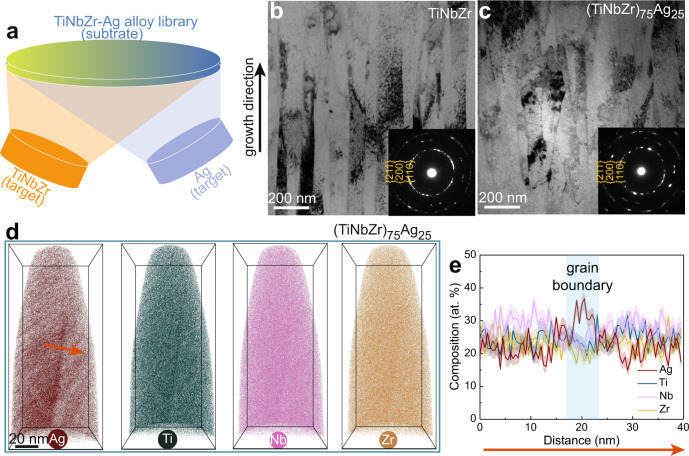
Fabrication and characterization of the TiNbZr-Ag alloys. **a** A schematic illustration of the combinatorial magnetron co-sputtering approach. **b**, **c** Side-view TEM images of the TiNbZr and (TiNbZr)_75_Ag_25_ alloys. The inset images show the corresponding SAED pattern. **d** Three-dimensional (3D) APT reconstruction showing elemental distribution in the (TiNbZr)_75_Ag_25_ alloy. **e** 1D compositional profile measured along the red arrow in (**d**), showing that the grain boundaries of the (TiNbZr)_75_Ag_25_ alloy are enriched with Ag.

We deposited (TiNbZr)_75_Ag_25_ (at.%) films on equiatomic TiNbZr alloy sheets using a combinatorial magnetron co-sputtering approach^25^. By varying the distance between the substrates and the sputtering targets, TiNbZr-Ag alloys with different Ag content were prepared in a single deposition (Fig. 1a). For comparison, we also investigated (TiNbZr)_90_Ag_10_ (at.%) and equiatomic TiNbZr alloy films. X-ray diffraction (XRD) spectra (Supplementary Fig. 1) suggest that the TiNbZr, (TiNbZr)_90_Ag_10_, and (TiNbZr)_75_Ag_25_ alloys assume a single-phase body centered cubic (bcc) structure. Transmission electron microscopy (TEM) shows that the (TiNbZr)_75_Ag_25_ alloy and the reference TiNbZr alloy are both composed of ~100-nm-diameter nanograins elongated along the growth direction (Fig. 1b, c). No secondary phase is observed in these alloys, as confirmed by selected area electron diffraction (SAED) analysis (inset in Fig. 1b, c). We further characterized the elemental distribution using atom probe tomography (APT) (Fig. 1d). The (TiNbZr)_75_Ag_25_ alloy has an average composition of Ti_25_Nb_27_Zr_23_Ag_25_ (at.%), and the grain boundaries are enriched with Ag. The grain boundary segregation is driven by a reduction in free energy following the Gibbs adsorption isotherm^26^. We observe composition striations within the grains (Supplementary Fig. 2). Ag has a Pauling electronegativity of 1.93, a value that is distinctly different from that of the other three elements (1.54 for Ti, 1.33 for Zr, and 1.60 for Nb)^27^. This difference might explain the underlying bonding effects behind the nanoscale chemical inhomogeneity observed in the material^28^.

### Mechanical properties and wear behavior

Alloying with Ag leads to the increase of the yield strength (*σ*_*y*_) from 1.1 ± 0.1 GPa for the TiNbZr alloy to 1.3 ± 0.2 GPa for the (TiNbZr)_90_Ag_10_ alloy and 1.7 ± 0.2 GPa for the (TiNbZr)_75_Ag_25_ alloy, as probed in uniaxial micropillar compression experiments (Supplementary Fig. 3). The increased strength can be attributed to the enhanced solid solution strengthening and the formation of composition striations via Ag addition, both increasing the friction stress that acts against dislocation motion. All three alloys reveal homogeneous deformation to compressive strain of 30%.

**Fig. 2:**
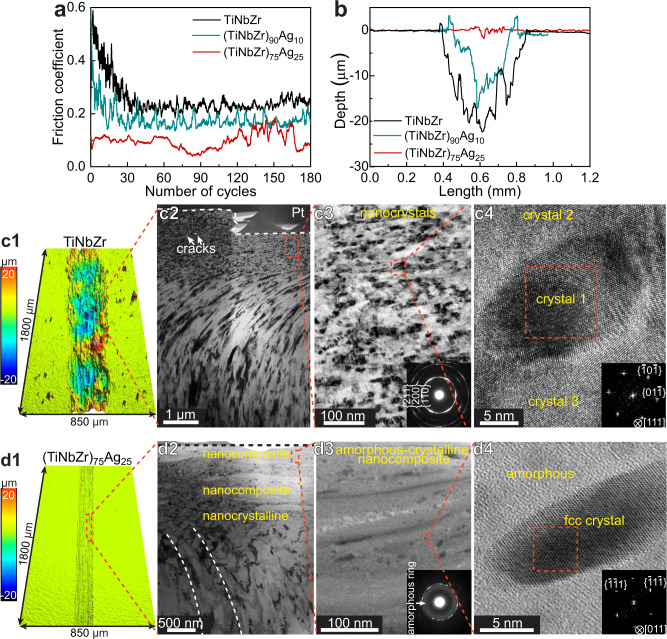
Wear behavior of the TiNbZr, (TiNbZr)_90_Ag_10_, and (TiNbZr)_75_Ag_25_ alloys. **a** Friction coefficients as a function of sliding cycles for the three alloys. **b** 2D cross-sectional profiles of the wear tracks. **c1** 3D profile of the TiNbZr wear surface. **c2**-**c4** TEM images revealing the grain refinement in the worn TiNbZr alloy. The TEM lamella was prepared from the edge of the wear track, as the TiNbZr alloy film was completely removed at the center of the wear track. **d1** 3D profile of the (TiNbZr)_75_Ag_25_ wear surface. **d2**-**d4** Structure of the worn (TiNbZr)_75_Ag_25_ alloy. **d2** TEM image presenting a ~400-nm-thick amorphous-crystalline nanocomposite on the surface. **d3** Annular bright field (ABF)-STEM image displaying the structure of the nanocomposite. **d4** Atom-resolved ABF-STEM image of the nanocomposite, presenting a fcc structured nanocrystal embedded in the amorphous matrix. The inset image in (**c3**) and (**d3**) are the corresponding SAED patterns, and the insets in (**c4**) and (**d4**) are the FFT patterns of the regions marked by the red rectangles.

The wear behavior of the TiNbZr, (TiNbZr)_90_Ag_10_, and (TiNbZr)_75_Ag_25_ alloys was studied by sliding them against stainless-steel balls (4-mm-diameter) in ambient air (Fig. 2). The sliding velocity was 1 mm/s, the slide stroke was 5 mm, and the contact force was 5 N, corresponding to a maximum Hertzian contact stress of 1.0 GPa. One sliding cycle corresponds to two strokes, and the sliding duration was 1800 s for 180 cycles. The TiNbZr alloy reveals a steady state friction coefficient of ~0.25, representing a typical constant friction observed in unlubricated sliding of metals^29^. The (TiNbZr)_90_Ag_10_ alloy exhibits a lower steady state friction coefficient of ~0.16, which is mainly attributed to the lubricating effect of Ag^30^. Notably, the (TiNbZr)_75_Ag_25_ alloy shows a low friction coefficient (~0.09) throughout the wear process (Fig. 2a). The stainless-steel ball maintains its spherical shape despite the high contact stress (Supplementary Fig. 4). We compared the morphology of the wear surfaces of the TiNbZr and (TiNbZr)_75_Ag_25_ alloys using a 3D optical microscope (Fig. 2b, c1, d1). The (TiNbZr)_75_Ag_25_ alloy shows a narrow and smooth wear track, which is profoundly different from the wide and deep wear track of the TiNbZr alloy. Hence, the (TiNbZr)_75_Ag_25_ alloy has a dramatically smaller wear volume (1.5 × 10^5^ μm^3^) as compared to the TiNbZr (3.0 × 10^7^ μm^3^) and (TiNbZr)_90_Ag_10_ (1.1 × 10^7^ μm^3^) alloys, corresponding to a two orders of magnitude reduction in specific wear rate (1.7 × 10^−5^ mm^3^ N^−1^ m^−1^) compared to the TiNbZr (3.3 × 10^−3^ mm^3^ N^−1^ m^−1^) and (TiNbZr)_90_Ag_10_ (1.2 × 10^−3^ mm^3^ N^−1^ m^−1^) alloys.

### Friction-induced amorphous-crystalline oxidic nanocomposite surface layer

To understand the wear mechanism, we investigated the structures of the wear surfaces at atomic scale using TEM and aberration-corrected scanning TEM (STEM). In the worn TiNbZr alloy, the original columnar grains have been refined into ~10-nm-diameter globular grains within ~1 μm depth from the surface (Fig. 2c2–c4). Grain refinement is a common phenomenon for crystalline metals exposed to wear. This effect is attributed to the high density of dislocations forming sub-grain boundaries to reduce their distortion energy^29^. The wear surface of the (TiNbZr)_90_Ag_10_ alloy shows a multilayer structure comprising regions of a ~20-nm-thick nanocrystalline phase separated by ~5-nm-thick amorphous interfaces (Supplementary Fig. 5). In the worn (TiNbZr)_75_Ag_25_ alloy, the nanograins are slightly curved at a depth of 1.6–3.4 μm below the surface, and globular grains with ~30 nm diameter are observed at 1.6 μm depth from the surface (Fig. 2d2). Different from the two reference alloys, a ~400-nm-thick amorphous surface layer, in which nanocrystals with diameters from 5 nm to 20 nm are embedded, is found in the worn (TiNbZr)_75_Ag_25_ alloy (Fig. 2d3). The nanocrystals have face centered cubic (fcc) structure (Fig. 2d4). Energy-dispersive X-ray spectroscopy (EDS) results indicate that the amorphous matrix and the nanocrystals are enriched with O and Ag, respectively (Supplementary Fig. 6). Therefore, the ~400-nm-thick surface layer of the worn (TiNbZr)_75_Ag_25_ alloy is identified as an amorphous-crystalline nanocomposite containing ~10 nm-sized Ag nanocrystals embedded in a TiNbZr-O amorphous oxide. Furthermore, many Ag nanocrystals contain nanotwins (Supplementary Fig. 7), which results from the low stacking fault energy (~16 mJ m^−2^) of Ag^31^.

**Fig. 3:**
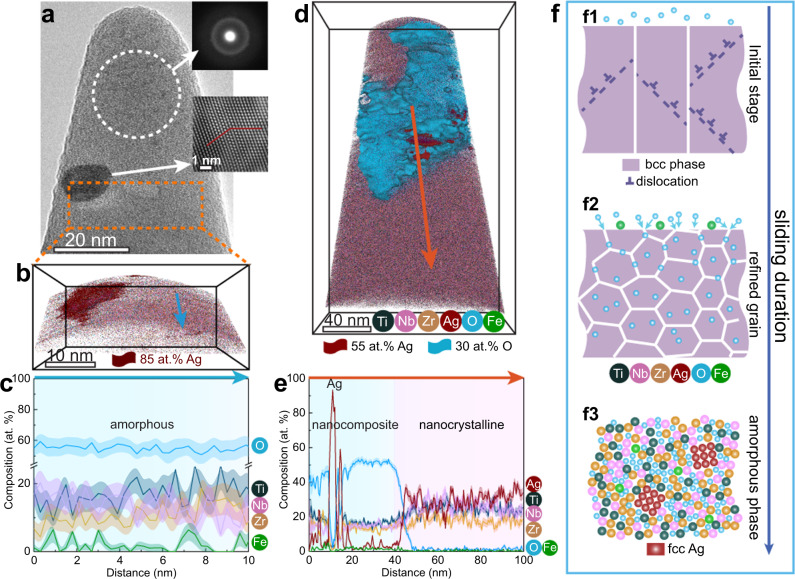
Formation mechanism of the amorphous-crystalline nanocomposite nanolayer on the wear surface of the (TiNbZr)_75_Ag_25_ alloy. **a** TEM image of the needle-shaped sample prepared for correlative TEM-APT investigation, revealing the material’s amorphous-crystalline structure. The inset at the top is a SAED pattern for the white dashed circle zone in (**a**), showing a halo ring pattern of the amorphous phase. The inset in the middle is a magnified image of the nanocrystal in (**a**), revealing a nanotwin feature. **b** 3D reconstruction of the APT dataset for the correlative TEM-APT sample. The Ag nanocrystal is highlighted by iso-composition surfaces encompassing regions containing more than 85 at.% Ag. **c** 1D compositional profile measured along the arrow indicated in (**b**). **d** 3D reconstruction of the APT sample prepared from the wear scar. The Ag and O enriched regions are highlighted using 55 at.% Ag and 30 at.% O iso-composition surfaces, respectively. **e** 1D compositional profile measured along the arrow marked in (**d**). **f** Schematic diagram illustrating the formation mechanism of the amorphous-crystalline oxide nanocomposite during wear. (**f1**) Plastic deformation via dislocation generation and motion; (**f2**) Grains are refined and the abundant grain boundaries accelerate the ingress of O; (**f3**) The massive amounts of O enhance the GFA of the alloy, promoting the formation of an amorphous-crystalline structure.

In order to unveil the formation mechanism of the amorphous-crystalline nanocomposite on the wear surface, we further performed correlative TEM-APT experiments on the nanocomposite surface to analyze the structural and chemical information also at near-atomic scale. Through correlative TEM investigation of an APT sample (Fig. 3a), prepared from the top surface of the wear track, we identified the nanocrystals and amorphous phase as Ag and Ti_17_Nb_13_Zr_13_Fe_2_O_55_ (at.%), respectively (Fig. 3b, c). Another APT analysis from the wear scar reveals three compositionally distinct regions (Fig. 3d, e), i.e., an amorphous region, Ag nanocrystals, and a nanocrystalline region. The nanocrystalline region has a composition (Ti_24_Nb_26_Zr_20_Ag_29_O_1_ (at.%)) close to that of the original material. The three compositionally distinct regions, revealed by APT probing, agree well with the same features observed by STEM-EDS characterization (Supplementary Fig. 6). During the wear process, plastic deformation is associated with the generation and movement of dislocations, confined inside the nanograins by the grain boundaries of the (TiNbZr)_75_Ag_25_ alloy (Fig. 3f1). The accumulation of a high density of dislocations leads to the formation of sub-grain boundaries^29^. Consequently, the originally 100-nm-wide columnar grains are refined into ~30-nm-diameter globular grains, shown in the nanocrystalline region in Fig. 2d2. The evolving high grain boundary density provides abundant pathways for the ingress of O^32^. Moreover, Ag is a highly active catalyst, promoting fast dissociation of atmospheric O_2_ and enabling adsorption of O^33^. The grain boundaries of the as-fabricated (TiNbZr)_75_Ag_25_ alloy are enriched with Ag, further accelerating this process. Therefore, massive amounts of O are blended into the alloy’s sub-surface region (Fig. 3f2). The empirical rules of glass-forming ability (GFA) state that an alloy containing three or more elements with large negative enthalpy of mixing and significant atomic size difference are prone to form a glass^24^. The incorporation of the small O atoms into the (TiNbZr)_75_Ag_25_ alloy modifies the atomic coordination with directed bond contributions and a large negative enthalpy of mixing^23^, thus enabling the in-situ formation of an amorphous oxide during wear. Although Ag has a relative large negative enthalpy of mixing with Zr and Ti (−20 kJ/mol and −2 kJ/mol, respectively), it has a positive enthalpy of mixing with Nb (+16 kJ/mol)^24^, which serves as a thermodynamic driving force to nucleate the Ag nanocrystals in the amorphous Ti_17_Nb_13_Zr_13_Fe_2_O_55_ matrix (Fig. 3f3).

## Discussion

**Fig. 4:**
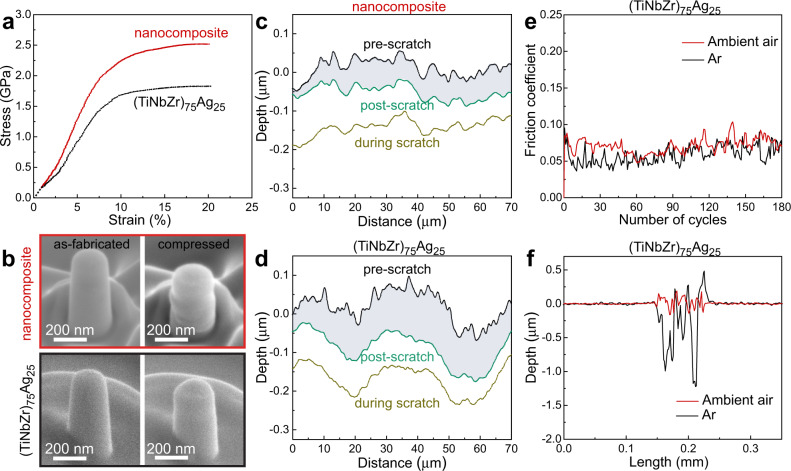
Effect of the amorphous-crystalline nanocomposite on wear reduction. **a** Typical compressive engineering stress-strain curves obtained for the 200-nm-diameter nanopillars of the nanocomposite and of the as-deposited (TiNbZr)_75_Ag_25_ alloy. **b** SEM images of the nanocomposite and the (TiNbZr)_75_Ag_25_ alloy nanopillars before and after compression. **c**, **d** Surface profiles as a function of scratch distance for the nanocomposite and (TiNbZr)_75_Ag_25_ alloy. **e** Friction coefficients of the (TiNbZr)_75_Ag_25_ alloy sliding against 4-mm-diameter stainless steel ball in Ar atmosphere and ambient air, respectively. **f** The corresponding 2D cross-sectional profiles of the wear tracks.

Amorphous materials generally deform via the activation and motion of collective atomic rearrangements, i.e., shear transformation zones (STZs)^34^. With the onset of plastic deformation, the STZs evolve into mature shear bands which propagate rapidly and lead to catastrophic failure. If the size of the amorphous regions is smaller than 100 nm, shear banding events can be suppressed, leading to high strength and homogeneous plastic flow^35^. It has been demonstrated that crystal-glass nanocomposite alloys, with solid solution nanocrystals embedded in the amorphous matrix, reveal homogeneous plastic deformation, due to the confined plastic flow behavior of the amorphous phase^22,36^. Similarly, the amorphous-crystalline nanocomposite generated in the current study during wear benefits from this dual-phase structure, due to the resulting homogeneous plastic flow. Hence, cracking and brittle failure are suppressed in the nanocomposite nanolayer. Shear bands are not observed in the worn (TiNbZr)_75_Ag_25_ alloy in SEM (Supplementary Fig. 8) and TEM/STEM (Fig. 2) observations, confirming homogeneous plastic flow of the material. In fact, the homogeneous plastic flow behavior of amorphous materials usually indicates that the flow stress approaches the theoretical strength regime^35^. Compared with conventional nanograined metals^37^, the amorphous phase with its theoretical strength effectively prevents softening that could have been potentially caused by the Ag nanocrystals and their coarsening. Therefore, the grain size of the Ag nanocrystals, spatially confined in the amorphous-crystalline nanocomposite nanolayer, remains unchanged along the through-thickness direction, and the material close to the surface sustains high strains^38^. Furthermore, the Ag nanocrystals reveal a higher probability for nanotwinning on the top surface of the wear track (Fig. 3a) and in the wear debris (Supplementary Fig. 7), which are regions that experience high local strains. The nanotwin boundaries not only provide effective barriers against dislocation motion but also serve as an additional independent shear carrier^39^, contributing to a high flow stress and deformability of the Ag nanocrystals. As a result, the amorphous-crystalline surface nanocomposite formed during the reactive wear process exhibits a compressive strength *σ*_*y*_ of 2.4 GPa that is 41% above that of the as-fabricated (TiNbZr)_75_Ag_25_ alloy, and reveals homogeneous deformation to a strain of 20% without catastrophic failure (Fig. 4a, b). The nano-scratch tests suggest that the nanocomposite reveals a wear volume that is 42% lower than that of the as-fabricated (TiNbZr)_75_Ag_25_ alloy (Fig. 4c, d).

We further compared the wear behavior of the (TiNbZr)_75_Ag_25_ alloy in Ar atmosphere and ambient air, respectively (Fig. 4e, f). The alloy tested in Ar and in ambient air has in both cases a comparable friction coefficient of ~0.08. However, in Ar atmosphere with little O, the reactive wear process is suppressed, which leads to an 11 times higher wear volume compared to the one measured in air. The oxidic nanocomposite in-situ formed in air has a *σ*_*y*_ that is 41% higher than that of the (TiNbZr)_75_Ag_25_ alloy, leading to a reduced contact area during wear. Moreover, the nanocomposite reveals homogeneous plastic deformation, preventing cracking or localized brittle fracture upon wear exposure. Therefore, once the oxidic nanocomposite is formed on the crystalline alloy’s surface during wear, its high strength and plastic deformability lead to a substantial improvement in wear resistance. The notable difference in wear volumes of the (TiNbZr)_75_Ag_25_ alloy upon wear in Ar and air proves the effective protection effect through the development of a friction-induced oxide nanocomposite surface. It works through exploiting the chemical interaction between the worn multi-component alloy and its oxidative environment, leading to excellent wear resistance, an effect referred to as reactive wear protection.

In order to reveal the controllability of the formation of the amorphous-crystalline nanocomposite, we evaluated the wear behavior of the (TiNbZr)_75_Ag_25_ alloy in ambient air for various sliding durations (180 cycles, 720 cycles, and 1800 cycles), sliding velocities (1 mm/s, 2 mm/s, and 4 mm/s), and contact forces (5 N, 10 N, and 15 N) (Supplementary Fig. 9). The increase of the sliding duration from 180 cycles to 1800 cycles (while the other wear conditions remained unchanged) leads to a reduction of the specific wear rate by 58%, from 1.7 × 10^−5^ mm^3^ N^−1^ m^−1^ to 7.0 × 10^−6^ mm^3^ N^−1^ m^−1^. TEM investigation reveals that the thickness of the amorphous-crystalline nanocomposite surface layer increases from 400 nm (Fig. 2d2) to 1.55 μm (Supplementary Fig. 10a1-a3) after increasing the sliding duration from 180 cycles to 1800 cycles. This observation suggests a dynamic formation process of the oxidic nanocomposite during wear, i.e., the high-strength nanocomposite is gradually thickened with ongoing sliding exposure. On the other hand, the increase of the sliding velocity or contact force (while the other wear conditions remained unchanged) leads to an increased wear rate of the (TiNbZr)_75_Ag_25_ alloy. A higher velocity shortens the time for the alloy’s surface to react with the environmental O during sliding, hindering the formation of an oxidic nanocomposite. Therefore, a thinner oxide nanocomposite layer with a thickness of only 115 nm is formed on the alloy’s surface after sliding with a velocity of 4 mm/s for 180 cycles (Supplementary Fig. 10b1-b3), resulting in a higher specific wear rate of 5.5 × 10^−5^ mm^3^ N^−1^ m^−1^. Similarly, a higher contact force of 15 N leads to deeper contact during wear, which induces a thinner nanocomposite layer with a thickness of 240 nm (Supplementary Fig. 10c1–c3) and a higher specific wear rate of 4.2 × 10^−5^ mm^3^ N^−1^ m^−1^. Although the thickness of the nanocomposite surface layer changes with the sliding condition, the amorphous-crystalline structure of the surface layer is identical for all testing conditions in the present work. This finding indicates that the formation mechanism of the amorphous-crystalline oxidic nanocomposite does not change with the sliding condition, confirming the intrinsic reactive wear protection ability of the (TiNbZr)_75_Ag_25_ alloy.

In summary, we propose a strategy to design wear-resistant alloys through in-situ formation of strong and deformable oxide nanocomposite surfaces during wear, a process which we refer to as ‘reactive wear protection’. A representative bcc structured (TiNbZr)_75_Ag_25_ (at.%) alloy was developed, which yields a two orders of magnitude lower wear rate than the reference TiNbZr alloy. Upon wear in ambient air, the (TiNbZr)_75_Ag_25_ alloy forms an in-situ 400-nm-thick amorphous-crystalline nanocomposite surface layer, through reaction with the environmental O, comprising an amorphous Ti_17_Nb_13_Zr_13_Fe_2_O_55_ matrix containing ~10-nm-sized Ag nanocrystals. The high wear resistance of the (TiNbZr)_75_Ag_25_ alloy is achieved by the synergistic effect of the low friction coefficient (~0.09) of the alloy and the in-situ formation of an amorphous-crystalline oxidic nanocomposite surface, which exhibits a high strength and homogeneous plastic flow behavior. The study provides a guideline to the future design of wear-resistant alloys, which is based on the formation of amorphous-crystalline nanocomposite surface following the reactive wear protection approach, i.e., by alloying the surface with environmental atoms during wear.

## Methods

### Materials fabrication

We prepared (TiNbZr)-Ag alloy films by magnetron co-sputtering of an equiatomic TiNbZr alloy target and an elemental Ag (99.99% purity) target. The alloy films were deposited on 1-mm-thick equiatomic TiNbZr cast alloy substrates. The substrates were polished to possess mirror-like surfaces prior to deposition. The varying distances between the substrates and the targets generated a series of (TiNbZr)-Ag alloys with different Ag content in a single deposition. The growth rate of the alloy films was controlled to be ~2.4 Å/s by manipulating the target power. The alloy films have a thickness of 4 μm. As a reference material, the TiNbZr alloy film was fabricated by sputtering with an equiatomic TiNbZr alloy target at a deposition rate of 2 Å/s. The base vacuum for sputtering was below 10^−7^ Torr, and the Ar pressure during sputtering was 0.3 Pa.

### Structure characterization

The structures of the TiNbZr, (TiNbZr)_90_Ag_10_, and (TiNbZr)_75_Ag_25_ alloy films were investigated using grazing incidence XRD (GIXRD) and transmission electron microscopy (TEM). GIXRD measurements were conducted using a Rikaku SmartLab diffractometer equipped with a Co K_α_ source. The incidence angle was 2°. TEM observations were carried out in a JEOL JEM-2200FS operated at 200 kV. Annular bright field (ABF)-scanning TEM (STEM) imaging was performed in a probe aberration-corrected STEM (FEI Titan Thermis) operated at 300 kV. The probe semi-convergence angle was 17 mrad, and the inner and outer semi-collection angles of the annular detector were from 13 to 21 mrad. TEM foils were prepared from the samples in a focused ion beam (FIB) workstation using Ga ions (FEI Helios Nanolab 600i). The final cleaning voltage/current was 5 kV/48 pA followed with 2 kV/23 pA.

### Atom probe tomography

The near-atomic scale elemental information of the as-deposited and worn (TiNbZr)_75_Ag_25_ alloy was obtained using a LEAP^TM^ 5000X HR (Cameca) under high vacuum of 2 × 10^−11 ^Torr. Atom probe tomography (APT) measurements of the as-deposited alloy were conducted in voltage mode with a pulse fraction of 15%, a pulse rate of 125 kHz, a target evaporation rate of 3 ions for 1000 pulses, and a specimen temperature of 60 K. The wear surface of the alloy was characterized in laser mode with a laser pulse energy of 40 pJ, a pulse rate of 125 kHz, and a specimen temperature of 60 K. The reconstruction of the 3D atom maps was carried out using the CAMECA integrated visualization and analysis software IVAS 3.8.6. Needle-shaped specimens for APT characterization were prepared using a site-specific FIB lift-out procedure^40^, and the final cleaning voltage/current was 2 kV/23 pA. For correlative TEM-APT investigation, the needle-shaped specimens were fabricated on a halved Mo TEM grid^41^ and subsequently analyzed in TEM (JEOL JEM-2200 FS) operated at 200 kV, followed by APT characterization.

### Mechanical properties

Nanoindentation tests were performed using a Hysitron TI700 nanoindenter with a Berkovich diamond tip. At least nine indentations were made on each alloy, and the indentation depth was kept within 10% of the film thickness. The elastic modulus is 85 GPa for the TiNbZr alloy, 85 GPa for the (TiNbZr)_90_Ag_10_ alloy, and 90 GPa for the (TiNbZr)_75_Ag_25_ alloy. The 1-μm-diameter micro-pillars and 200-nm-diameter nano-pillars for compression were fabricated from the samples using the FIB workstation. The final milling parameters were 30 kV and 10 pA. The taper angle of each pillar was carefully kept below 1.5°. The aspect ratio (height/diameter) was around 2. The pillar diameter was determined at 20% pillar height to avoid overestimation of strength due to taper angle. The compression experiments were performed in a nanoindenter (Keysight G200) with a diamond punch in load-control mode at a nominal strain rate of 5 × 10^−3^ s^−1^. Each compression experiment was conducted for at least five times to ensure repeatability.

### Wear performance analysis

The wear behavior of the TiNbZr, (TiNbZr)_90_Ag_10_, and (TiNbZr)_75_Ag_25_ alloy films deposited on TiNbZr substrates was evaluated by ball-on-disk reciprocating-sliding experiments using a Bruker UM II wear tester. The sliding tests were conducted under dry contact conditions in ambient air or in Ar atmosphere. 4-mm-diameter 316 L stainless steel balls were used to slide against the samples. The slide stroke was 5 mm. The elastic modulus of 316 L stainless steel is 193 GPa. The frictional force (*F*) was recorded by the wear tester automatically, and the friction coefficient was calculated as *F/P*, *P* is the contact force. The 3D profiles of the wear tracks were measured using a Wyko NT8000 optical profiler (Veeco Instruments Inc.).

The nanoscale wear behavior of the (TiNbZr)_75_Ag_25_ alloy film and the amorphous-crystalline nanocomposite was evaluated in the nanoindenter (Keysight G200) using a 10-μm-radius spherical diamond tip. The scratch force was 5 mN and the scratch distance was 80 μm. The surface morphology of the alloy was characterized in scanning mode using the same diamond tip with 0.1-mN-force before and after scratching. The data of 5 μm to the left and to the right was omitted to avoid border effect.

## Supplementary information

Supplementary Information

## Data Availability

All relevant data supporting the findings of this study are contained in the paper and its Supplementary Information files. All other relevant data are available from the corresponding authors (C.L., G.W. and D.R.) upon request.
