# Supplementary material for: Reactive wear protection through strong and deformable oxide nanocomposite surfaces
Source: Nat Commun. 2021 Sep 17;12:5518. doi: 10.1038/s41467-021-25778-y (PMC8448869; doi:10.1038/s41467-021-25778-y)
Supplement: Supplementary file 1 — Supplementary Information [file 41467_2021_25778_MOESM1_ESM.pdf]

Supplementary Information for

Reactive wear protection through strong and deformable oxide nanocomposite surfaces

Chang Liu*, Zhiming Li, Wenjun Lu, Yan Bao, Wenzhen Xia, Xiaoxiang Wu, Huan Zhao, Baptiste Gault, Chenglong Liu, Michael Herbig, Alfons Fischer, Gerhard Dehm, Ge Wu*, Dierk Raabe*

*Correspondence to: chang.liu@mpie.de (C.L.), ge.wu@mpie.de (G.W.), d.raabe@mpie.de (D.R.)

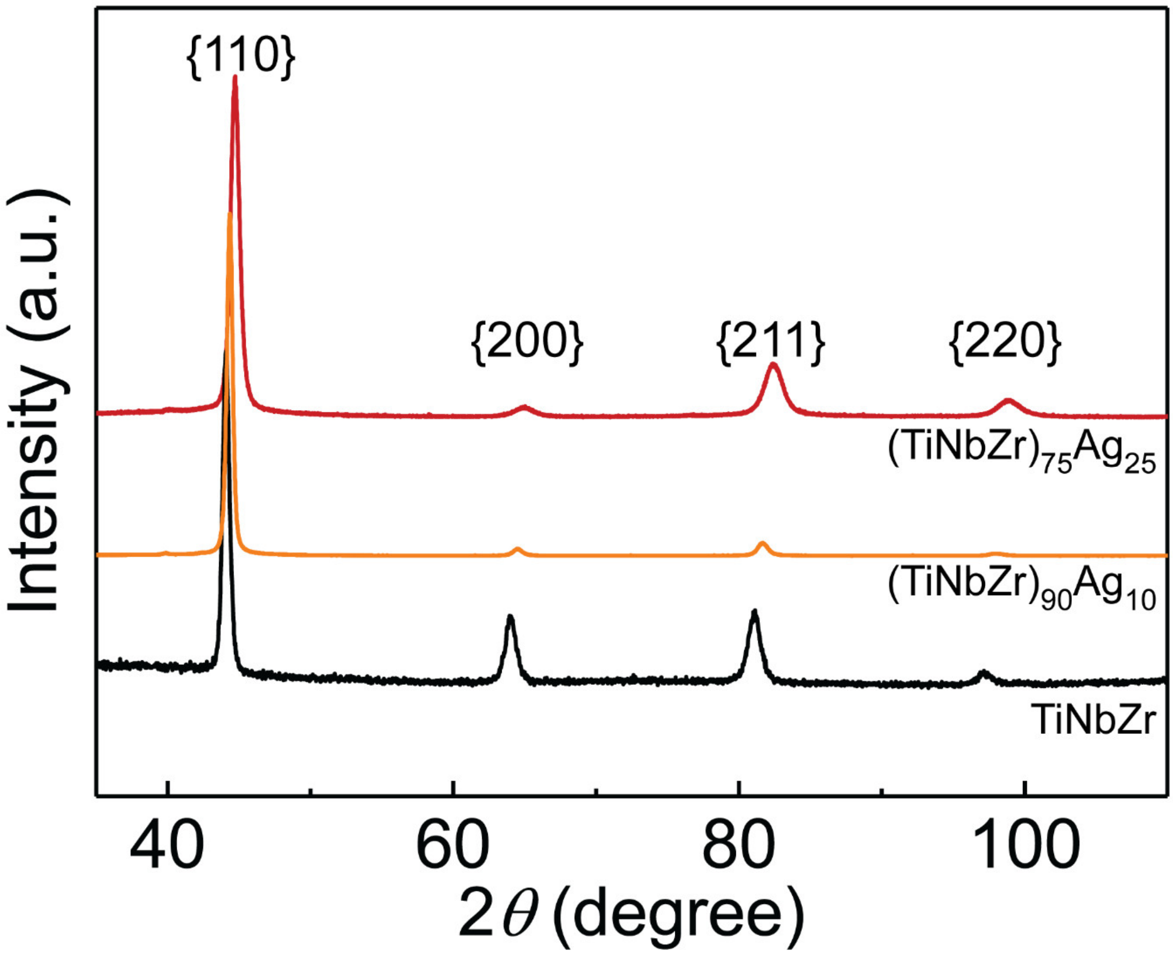

Supplementary Fig. 1 XRD spectra of the TiNbZr, $(TiNbZr)_{90}Ag_{10}$, and $(TiNbZr)_{75}Ag_{25}$ alloys, revealing single-phase bcc structure. The lattice constant decreases from 3.38 Å of TiNbZr alloy to 3.35 Å of $(TiNbZr)_{90}Ag_{10}$ alloy and 3.33 Å of $(TiNbZr)_{75}Ag_{25}$ alloy.

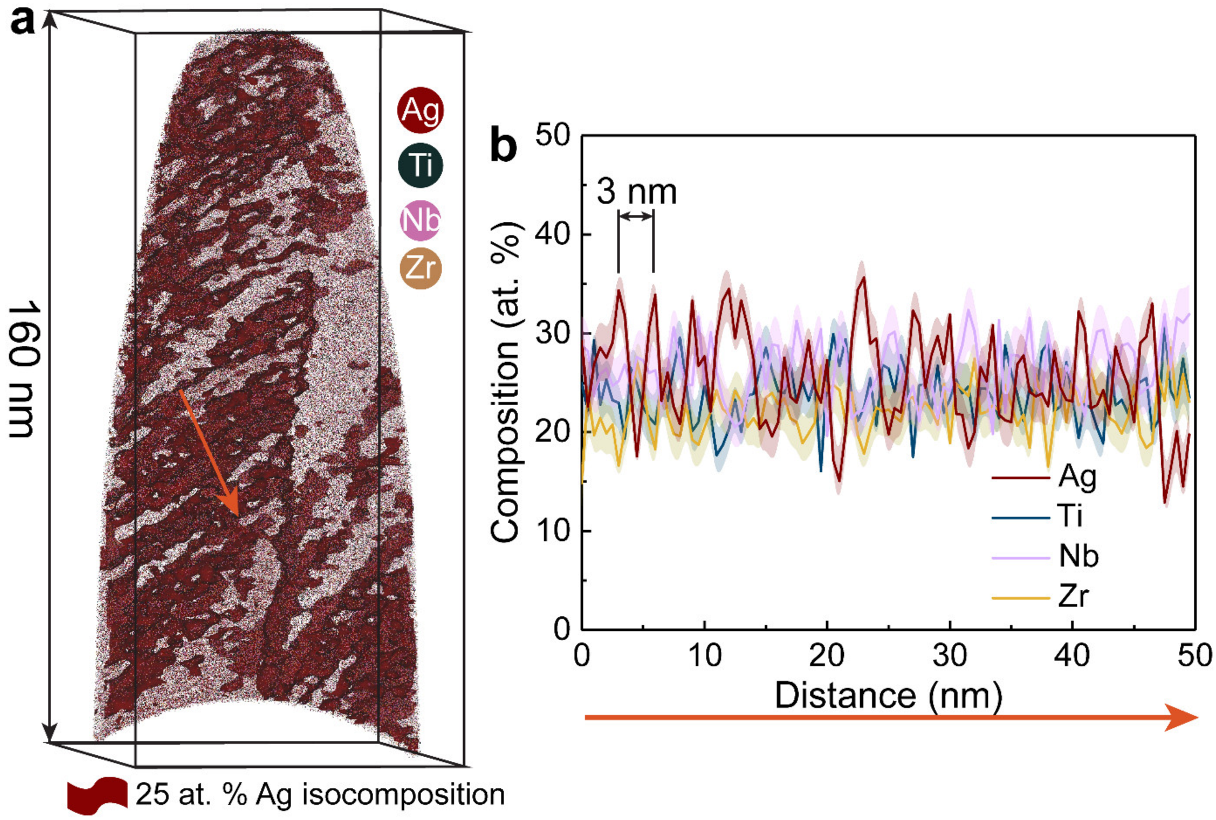

Supplementary Fig. 2 APT characterization of the $(TiNbZr)_{75}Ag_{25}$ alloy. a 10-nm-thick slice taken from the 3D reconstructed APT data, the Ag-enriched regions are highlighted with iso-composition surfaces with a threshold value of 25 at.% Ag. **b** 1D compositional profile measured along the red arrow shown in **(a)**, revealing composition striations with ~3-nm-spacing within the grains.

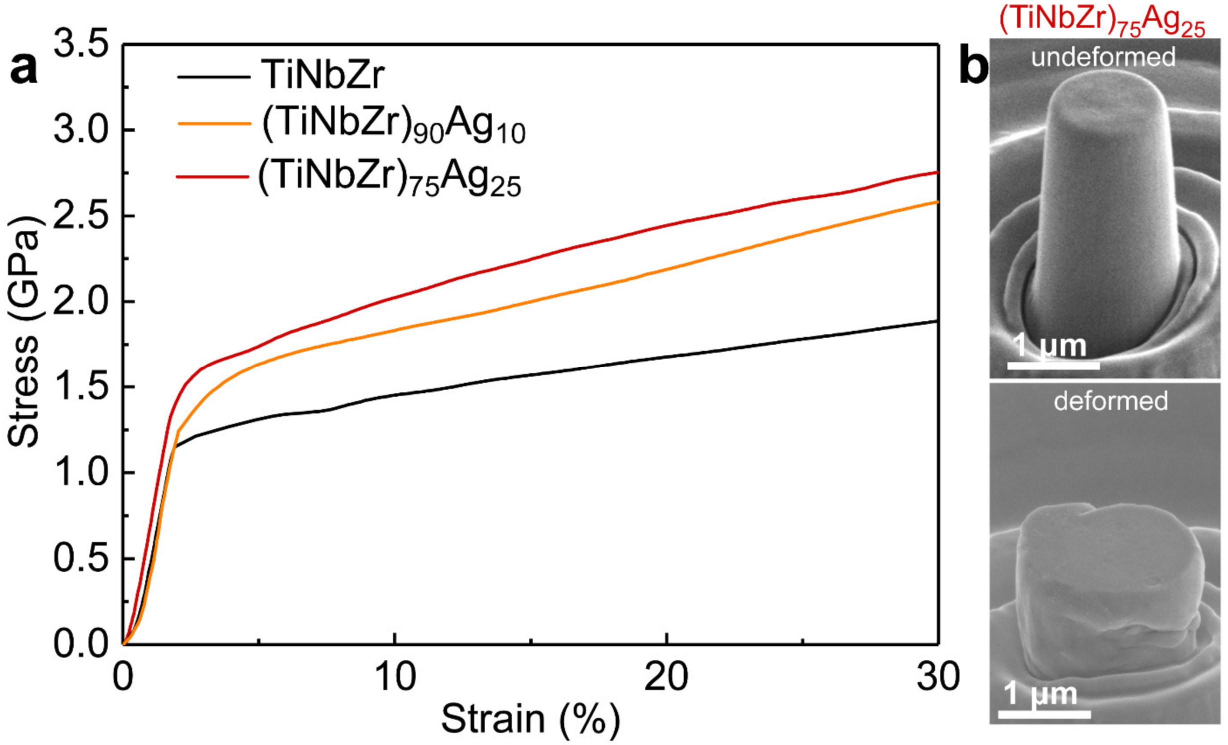

Supplementary Fig. 3 Mechanical properties of the TiNbZr, $(TiNbZr)_{90}Ag_{10}$, and $(TiNbZr)_{75}Ag_{25}$ alloys in compression. The 1-μm-diameter pillars were tested at a nominal strain rate of 5×10^{-3} s^{-1}. **a** Engineering stress-strain curves of the alloys. **b** SEM images presenting the pillar morphology of the $(TiNbZr)_{75}Ag_{25}$ alloy before and after compression.

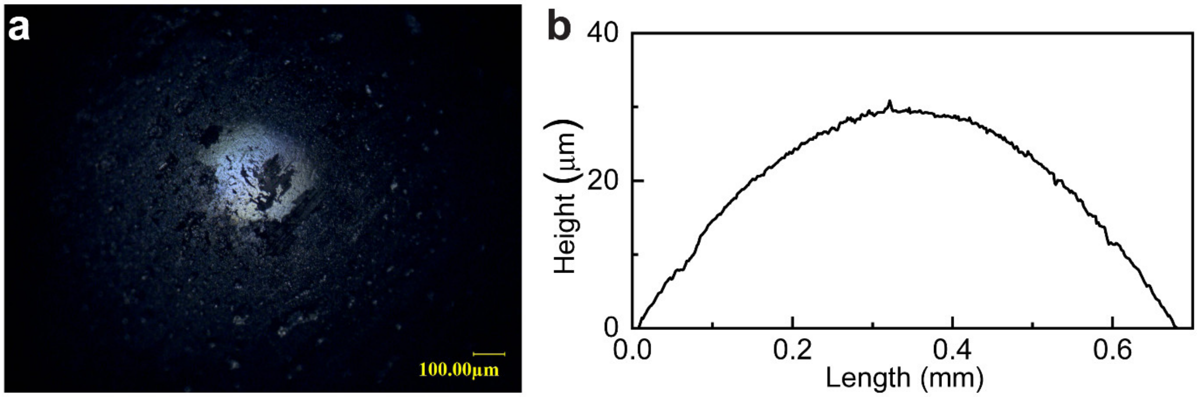

Supplementary Fig. 4 Surface morphology of stainless-steel counterbody after sliding against the $(TiNbZr)_{75}Ag_{25}$ alloy. a Optical micrograph presenting the wear scar of the stainless-steel ball. **b** 2D profile of the steel counterbody obtained using an optical profiler, showing that the spherical shape is preserved and there is unmeasurable wear for the ball.

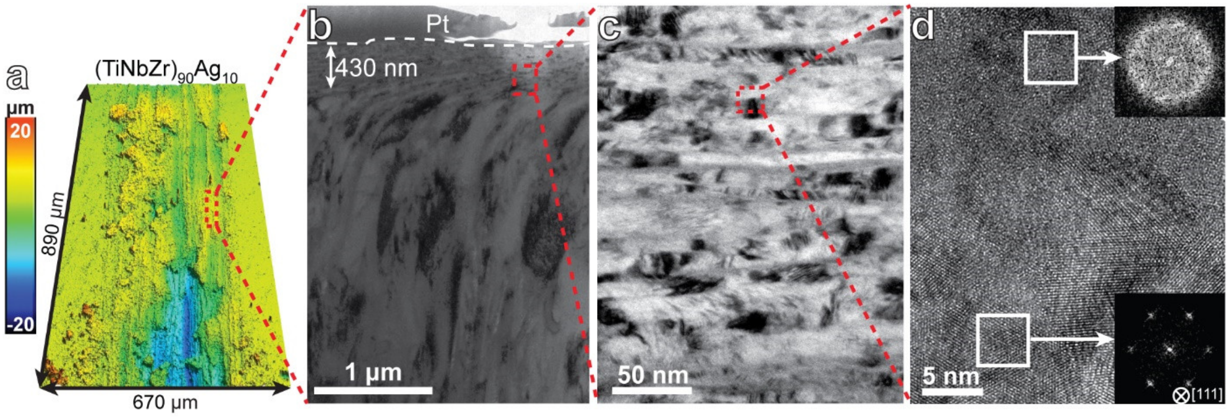

Supplementary Fig. 5 Wear track analysis of the $(TiNbZr)_{90}Ag_{10}$ alloy. a 3D profile of the wear surface. **b** TEM image presenting the structure of the worn $(TiNbZr)_{90}Ag_{10}$ alloy, a 430-nm-deep layer containing refined grains was formed at the surface. The TEM lamella was prepared from the edge of the wear track, as the $(TiNbZr)_{90}Ag_{10}$ alloy film was completely removed at the center of the wear track. **c** A magnified TEM image of the surface layer, revealing a multilayer structure comprising mainly nanocrystalline phase. **d** HRTEM image indicating that the nanocrystalline layers are separated by amorphous interfaces. The insets are the corresponding FFT patterns of the amorphous interface (top) and the nanocrystal (bottom).

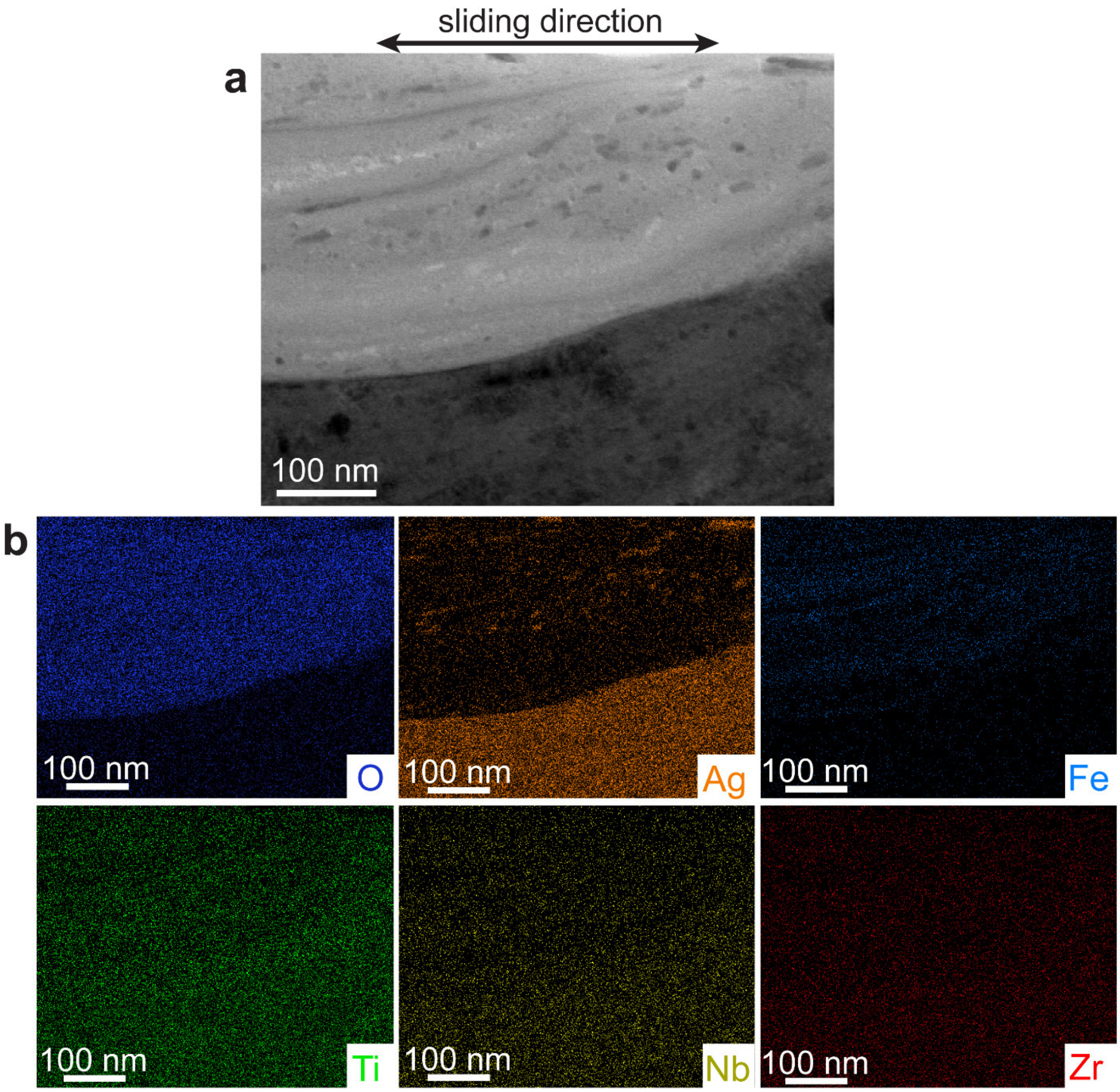

Supplementary Fig. 6 STEM imaging and elemental distribution mapping of the subsurface in the $(TiNbZr)_{75}Ag_{25}$ alloy after wear. a STEM image presenting the amorphous-crystalline nanocomposite on top of the nanocrystalline layer. **b** The corresponding EDS maps for individual elements of O, Ag, Fe, Ti, Nb, and Zr. The amorphous matrix contains O and Fe, which are not observed in the nanocrystalline layer. Fe from stainless steel counterbody intrudes upon wear. Besides, Ag-rich particles are embedded in the O-rich matrix.

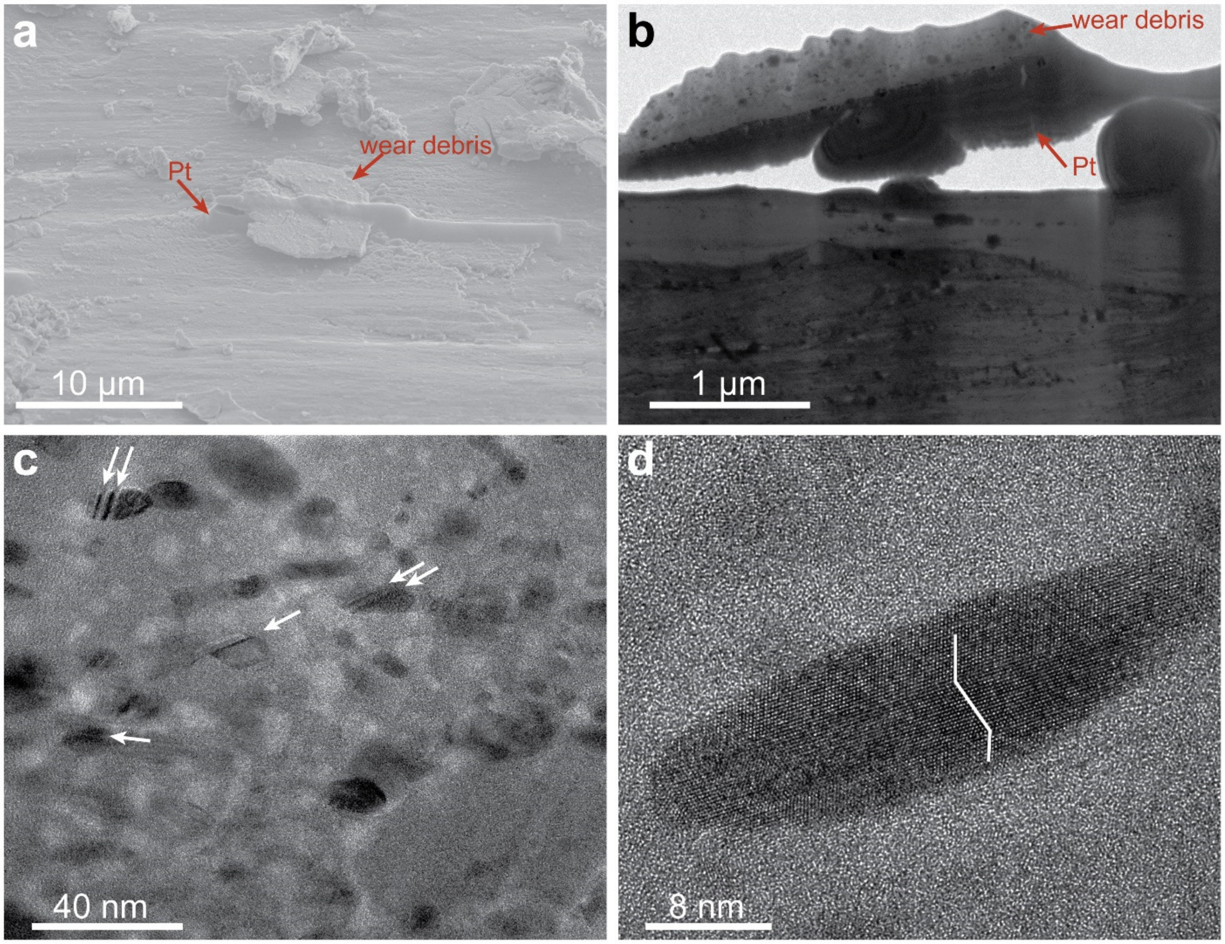

Supplementary Fig. 7 TEM characterization of the wear debris on the $(TiNbZr)_{75}Ag_{25}$ alloy, revealing a high amount of twinned Ag nanocrystals. a SEM image indicating the original position of the wear debris prepared for TEM observation. The Pt was deposited to protect the wear debris. **b** TEM image showing the wear debris. Note that an amorphous-crystalline nanocomposite layer with a thickness of ~400 nm is present beneath the wear debris. This confirms that the nanocomposite layer is generally observed on the wear surface of the $(TiNbZr)_{75}Ag_{25}$ alloy. **c** TEM image presenting that the wear debris has an amorphous-crystalline dual-phase structure, containing abundant twinned nanocrystals (indicated by the white arrows). **d** High resolution TEM (HRTEM) image showing a twinned Ag nanocrystal embedded in the amorphous matrix (marked by the white line).

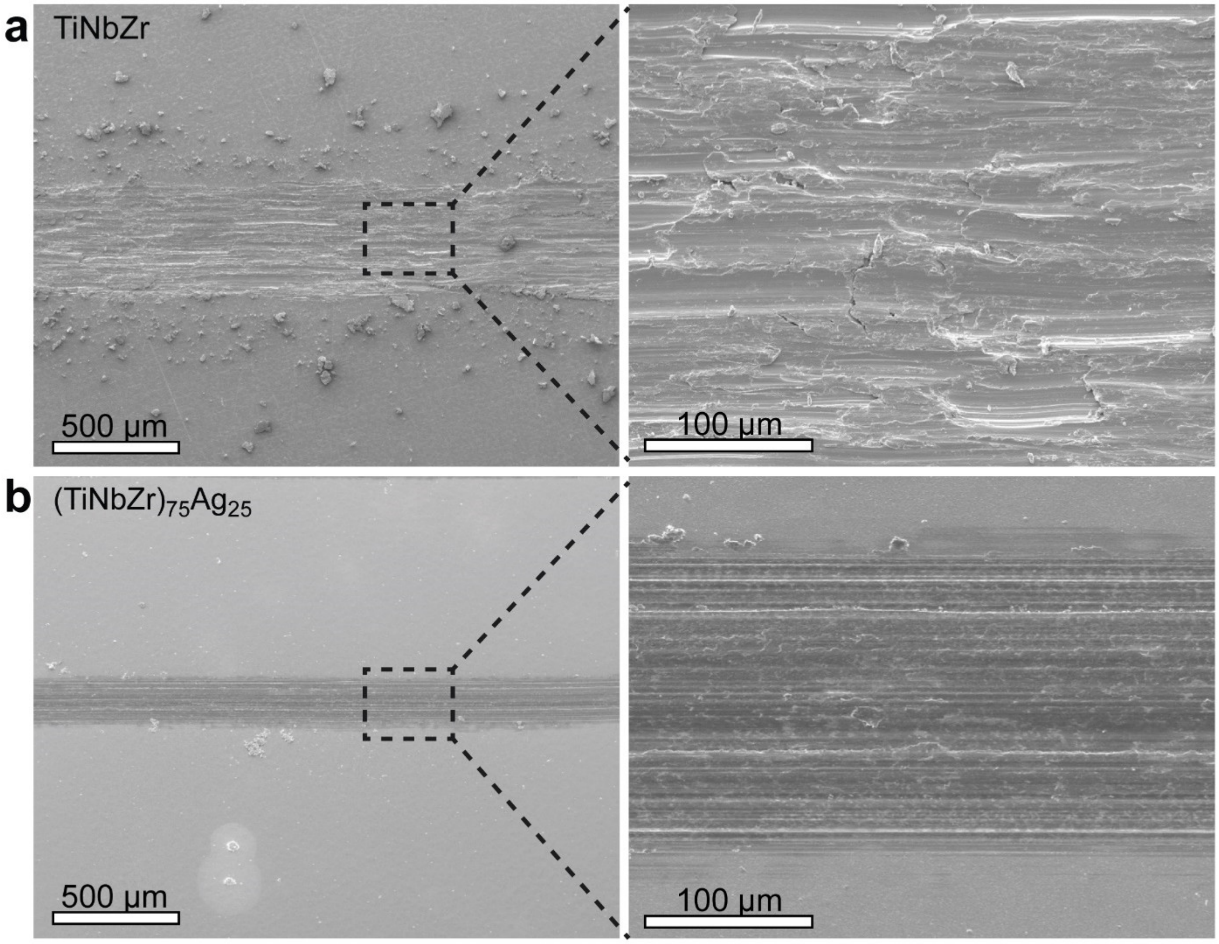

Supplementary Fig. 8 SEM images of wear surfaces of the TiNbZr and $(TiNbZr)_{75}Ag_{25}$ alloys. a Wear track of the TiNbZr alloy, revealing a rough surface with many cracks and wear debris. **b** $(TiNbZr)_{75}Ag_{25}$ wear track, showing a smooth surface without formation of cracks and shear bands.

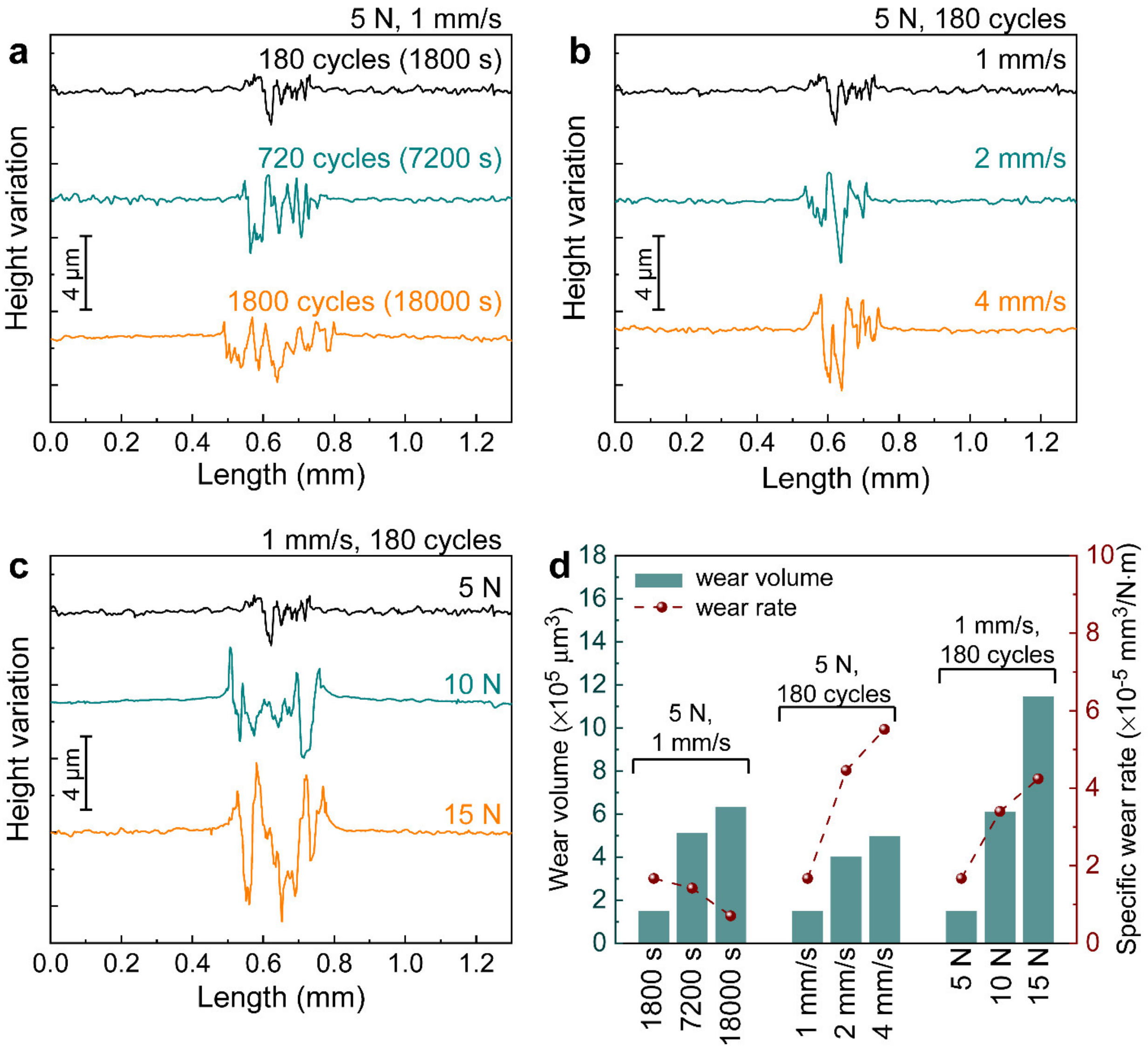

Supplementary Fig. 9 Wear behavior of the $(TiNbZr)_{75}Ag_{25}$ alloy upon testing with varying wear conditions. a-c 2D cross-sectional profiles of the wear tracks on the alloy after sliding with varied conditions. **d** Wear volume and specific wear rate of the $(TiNbZr)_{75}Ag_{25}$ alloy under various wear conditions.

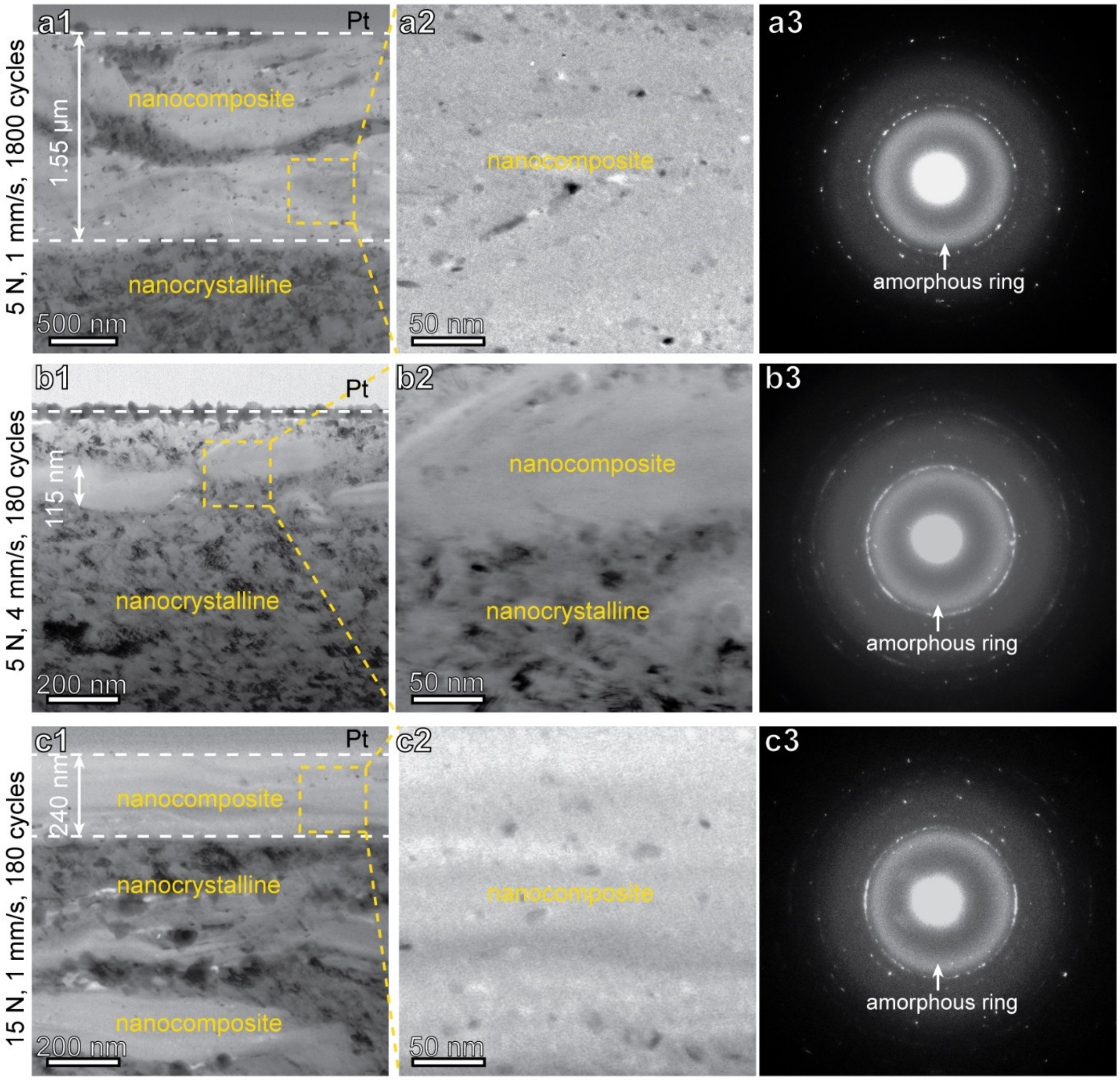

Supplementary Fig. 10 Structure of the worn (TiNbZr)$_{75}$Ag$_{25}$ alloy upon testing with different sliding conditions. a1 TEM image presenting a 1.55 μm-thick nanocomposite layer on the alloy's surface after sliding for 1800 cycles. **a2** Magnified TEM image showing the structure of the nanocomposite. **a3** SAED pattern of the nanocomposite displayed in (**a2**). **b1** TEM image showing that the alloy's surface is not yet fully covered with the nanocomposite after sliding with a velocity of 4 mm/s for 180 cycles. **b2** Magnified TEM image displaying the nanocomposite and

the adjacent nanocrystalline regions. **b3** SAED pattern of the area shown in (**b2**). **c1** TEM image presenting a 240 nm-thick nanocomposite layer on the surface of the worn alloy. **c2** A magnified TEM image of the nanocomposite. **c3** SAED pattern of the nanocomposite shown in (**c2**).
